# Radiomics Nomogram Improves the Prediction of Epilepsy in Patients With Gliomas

**DOI:** 10.3389/fonc.2022.856359

**Published:** 2022-03-30

**Authors:** Bai Jie, Yang Hongxi, Gao Ankang, Wang Yida, Zhao Guohua, Ma Xiaoyue, Wang Chenglong, Wang Haijie, Zhang Xiaonan, Yang Guang, Zhang Yong, Cheng Jingliang

**Affiliations:** ^1^ Department of Magnetic Resonance (MR), The First Affiliated Hospital of Zhengzhou University, Zhengzhou, China; ^2^ Shanghai Key Laboratory of Magnetic Resonance, East China Normal University, Shanghai, China

**Keywords:** radiomics, nomogram, glioma, epilepsy, MRI, imaging signs

## Abstract

**Purpose:**

To investigate the association between clinic-radiological features and glioma-associated epilepsy (GAE), we developed and validated a radiomics nomogram for predicting GAE in WHO grade II~IV gliomas.

**Methods:**

This retrospective study consecutively enrolled 380 adult patients with glioma (266 in the training cohort and 114 in the testing cohort). Regions of interest, including the entire tumor and peritumoral edema, were drawn manually. The semantic radiological characteristics were assessed by a radiologist with 15 years of experience in neuro-oncology. A clinic-radiological model, radiomic signature, and a combined model were built for predicting GAE. The combined model was visualized as a radiomics nomogram. The AUC was used to evaluate model classification performance, and the McNemar test and Delong test were used to compare the performance among the models. Statistical analysis was performed using SPSS software, and p < 0.05 was regarded as statistically significant.

**Results:**

The combined model reached the highest AUC with the testing cohort (training cohort, 0.911 [95% CI, 0.878–0.942]; testing cohort, 0.866 [95% CI, 0.790–0.929]). The McNemar test revealed that the differences among the accuracies of the clinic-radiological model, radiomic signature, and combined model in predicting GAE in the testing cohorts (p > 0.05) were not significantly different. The DeLong tests showed that the difference between the performance of the radiomic signature and the combined model was significant (p < 0.05).

**Conclusion:**

The radiomics nomogram predicted seizures in patients with glioma non-invasively, simply, and practically. Compared with the radiomics models, comprehensive clinic-radiological imaging signs observed by the naked eye have non-discriminatory performance in predicting GAE.

## Introduction

Glioma-associated epilepsy (GAE) is a common neurological symptom of glioma patients. The frequency of epilepsy in glioblastoma (60%) is lower than that in low-grade glioma (LGG) (89%); however, the incidence of glioblastoma is higher than that of LGG (1–3). Thus, the same focus is needed for predicting seizures in patients with glioblastoma. GAE greatly impairs patients’ quality of life, and if they do not receive treatment, some of them could develop status epilepticus and multiple seizures without regaining consciousness (4). Early prediction, increased awareness, and proper treatment and care are vital for protecting neurocognition, limiting progression, and improving patient quality of life (1, 5–7).The occurrence of GAE is multifactorial, including tumor location and microenvironment, peritumoral edema, alteration of the peritumoral environment, and genetic background. A tool for extracting and selecting massive amounts of tumor information, and offering a comprehensive and effective model for predicting the development of GAE in WHO grade II~IV gliomas, is needed.

Radiomics is a high-throughput method that extracts a large number of quantitative features from medical images and identifies features related to the target information in an objective, repeatable, and non-invasive way. Given these strengths, radiomics is widely used in differential diagnosis and prognostic assessment in clinical research, especially in studies of tumors (8–11). Glioma radiomics may be used to classify glioma grade, subtype, and gene type and to predict tumor proliferation, patient prognosis, etc., and is thus an area of high research interest (12–19). Magnetic resonance imaging (MRI) is an essential preoperative examination for patients with glioma due to its advantages in visualizing the central nervous system. MRI sequences selected for glioma radiomics research are mostly based on conventional sequencing (12–19), but multi-sequence MRI could be used to obtain a greater number of tumor features. However, previous studies have suggested that a single MRI sequence can achieve satisfactory results in glioma research (13, 14, 19–21), while multi-sequence MRI radiomics may increase the chances of overfitting. Radiomics based on a single MRI sequence has been shown to have effective performance in predicting the occurrence and type of GAE in patients with glioma (19–22). Radiomics nomogram is a simple and practical tool for assisting clinicians in performing differential diagnosis and is currently used in studies of epilepsy associated with LGG (19–21).

Therefore, the present study was designed to investigate the association between clinic-radiological features and GAE and develop and to validate a radiomics nomogram for predicting GAE in cerebral WHO grade II~IV gliomas.

## Materials and Methods

### Patients and MRI

**Figure 1:**
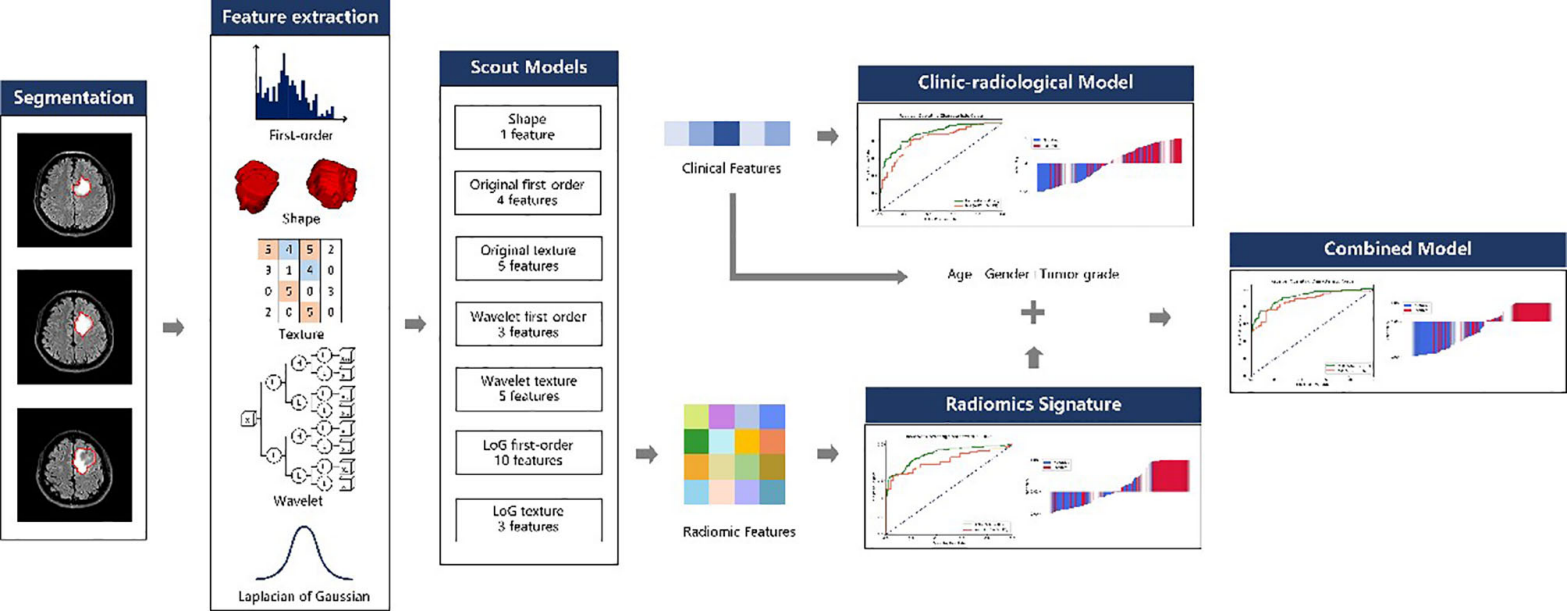
Radiomics workflow.

This retrospective study was approved by the Ethics Committee of Scientific Research and Clinical Experiments of the First Affiliated Hospital of Zhengzhou University, and the requirement for a written consent was waived. We enrolled 380 consecutive glioma patients who underwent MRI scanning before surgery at our hospital from August 2016 through August 2019. The inclusion criteria for all enrolled patients were (a) pathologically confirmed glioma (WHO grades II~IV) according to the 2016 World Health Organization central nervous system classification and (b) presurgical convention MRI scanning including T1-weighted, T2-weighted, diffusion-weighted, and T2 fluid-attenuated inversion-recovery (FLAIR) imaging. The exclusion criteria were as follows: (a) puncture biopsy and antitumor therapy initiation before MRI scan; (b) other lesions that could cause epilepsy, such as cerebral hemorrhage, stroke, and other brain tumors; and (c) WHO grade I glioma and non-singular tumors. The preoperative diagnosis of GAE was made based on clinical signs, electroencephalography (EEG), and imaging findings (23). All patients’ clinical characteristics including age, sex, tumor grade, and tumor location, and radiological characteristics including edema degree, cystic components, necrosis, hemorrhage (**Appendix 1**), crossing midline, and involvement of the cortex, were recorded. The whole workflow is illustrated in **Figure 1**.

MR images of patients scanned on 3.0-T MRI scanners (Magnetom Trio TIM/Prisma, Verio, or Skyra, Siemens Healthcare; Discovery 750, GE Medical Systems) were retrieved from the Picture Archive and Communication System at the hospital. The sequence acquisition parameters were as follows: T2 FLAIR images: matrix 256 × 256-pixel; field of view 240 × 240 mm; TI = 2,400–2,500 ms; TE = 81–135 ms; TR = 8,000–8,500 ms; section thickness = 5 mm and intersection gap = 1 mm. T2-weighted turbo spin-echo images (TR/TE 2,000–3,800/90–120 ms), and T1-weighted spin-echo images (TR/TE 200–220/2–3 ms) with sagittal and axial non-enhanced sequences, had a 256 × 256-pixel matrix and a 240 × 240-mm field of view, with section thickness = 5 mm and intersection gap = 1 mm. Diffusion-weighted images were obtained by using single-shot spin-echo echo-planar sequences (TR/TE 2,000–4,000/50–80), matrix 256 × 256; field of view 240 × 240 mm, section thickness 5 mm, intersection gap 1 mm, and b values = 0 and 1,000 s/mm^2^. Maps of the apparent diffusion coefficients (ADCs) were also generated.

### Tumor Masking and Image Preprocessing

A volume of interest (VOI) containing the entire tumor and peritumoral edema was manually drawn slice by slice on the T2 FLAIR images using ITK SNAP (version 3.6.0; www.itksnap.org) software by a PhD candidate in imaging for medicine (GA, 5 years of experience in neuro-oncology). Next, the segmentation results were reviewed and modified, if necessary, by a neuroradiologist with 20 years of experience in neuroradiology (BJ) using the same software. All images were normalized to a [0,1] range before feature extraction.

### Radiomics Feature Extraction

Feature extraction was performed with the PyRadiomics (version 3.0) (24) package in Python (3.7.6). For each patient, 3D shape features (n = 14) were extracted from the VOI. First-order statistical features (n = 18) and texture features (n = 75) were extracted from each of the following image types: (1) original T2 FLAIR images; (2) each of the three Laplacian-of-Gaussian (LoG) filtered images (sigma = 1.0, 3.0, 5.0); and (3) each of the eight 3D wavelet transformed image sub-bands using the Harr wavelet. The texture features extracted in this study included features based on the gray-level co-occurrence matrix (GLCM), gray-level run length matrix (GLRLM), gray-level size zone matrix (GLSZM), neighboring gray tone difference matrix (NGTDM), and gray-level dependence matrix (GLDM). A total of 1130 features were extracted for each patient.

### Feature Selection and Radiomic Signature Building

To remove the imbalance in the training dataset, upsampling was performed by repeating random non-GAE patient data.

A total of 1,130 radiomic features were extracted for each patient; however, dealing directly with so many features would limit the robustness and effectiveness of the model. Thus, we used a heuristic approach to reduce the feature dimensions and select the appropriate features for radiomics model building. First, all features were divided into seven subgroups according to their category, such as shape, first order, and texture. For each subgroup, a scout model was built with features in the subgroup using the training dataset. The best scout model for each subgroup was selected according to its performance in 10-fold cross-validation over the training cohort. When the optimal scout model’s area under the receiver operating characteristic curve (AUC) on cross-validation was >0.6, all features in the model were used for the final model building. Otherwise, all features in the subgroup were discarded.

To build the scout model, all features were normalized to the range of [0, 1]. Thereafter, Pearson correlation coefficient (PCC) values were calculated between all feature pairs, and if the PCC value between two features was >0.99, one of them was removed. To determine the best number of features to be retained in the model, three feature selectors were compared: recursive feature elimination (RFE), which repeatedly builds the model and eliminates the least important feature each time; relief, which calculates a feature score for every feature and ranks them accordingly; and the Kruskal–Wallis test (KW), which eliminates the most likely feature from the same distribution in both GAE and non-GAE samples. For the classifier, we compared the performance of the linear support vector machine (SVM), logistic regression (LR), and random forest (RF) classifiers due to their good interpretability and performance in diagnosis based on medical imaging.

To find the best model for each subgroup, we tested different combinations of feature selectors and classifiers. Therefore, nine models (3 feature selectors and 3 classifiers) were built for the scout models, and the one with the best cross-validation AUC was used. Multimodel building and comparison were implemented semiautomatically with the open-source software, FeAtureExplorer (FAE, version 0.4.0) (25), which uses scikit-learn (version 0.23.2) as the backend for machine learning.

As mentioned above, the features retained in the qualified scout models were used to build a radiomics model. The process of model building for radiomics was similar to that used in scout model building, but without PCC-based dimension reduction, due to the relatively small number of input features.

### Development of the Clinic-Radiological Model

Clinical characteristics including sex, age, and tumor grade were studied. The semantic radiological characteristics were assessed by a radiologist (BJ) with 15 years of experience and included hemorrhage (yes or no), cystic components (yes or no), necrosis (yes or no), edema degree (no/slight/middle/obvious), tumor location (left or right hemisphere, frontal, occipital, parietal, temporal, insular, thalamus, lateral ventricle or multilobe), and tumor components involving the cerebral cortex (edema/tumor/both/none). We established a clinical model using only clinic-radiological signs for distinguishing GAE from non-GAE.

### Development of the Radiomics Nomogram

Incorporating the radiomics signature and characteristics in the clinic-radiological model (age, sex, and tumor grade), a combined model was built using the logistic regression method. To provide clinicians with an individualized and easy-to-use tool for the preoperative prediction of the occurrence of epilepsy in glioma patients, the combined model was visualized as a radiomics nomogram.

### Performance Evaluation of the Models

The receiver operating characteristic (ROC) curve and AUC were used to evaluate the classification performance of the models for each cohort. The accuracy, sensitivity, specificity, positive predictive value (PPV), and negative predictive value (NPV) were also calculated at a cutoff value that maximized the value of the Youden index in the training cohort. The calibration curves and decision curve analyses (DCAs) (26) were used to assess the clinical usefulness of the clinic-radiological model, radiomic signature, and combined model. Calibration curves and the Hosmer–Lemeshow test were used to assess the agreement between the nomogram prediction probabilities of the GAE and the actual outcomes. To assess the performance difference among during the radiomics model, nomogram, and clinic-radiological model, the DeLong test and McNemar tests were performed.

### Statistical Analysis

To illustrate differences in the location of tumors for GAE and non-GAE patients, we superimposed the segmented VOIs of all patients in a group to obtain a 3D frequency map. Each map shows the frequency of occurrence of tumors at the voxel level in the template space. We then color-coded the frequency for visualization using Python (version: 3.7.6).

Age is reported as the mean and range, and its difference between the GAE and non-GAE groups was assessed by a two-sided t-test. The other clinical and radiological characteristics are reported as frequencies and proportions, and differences between the GAE and non-GAE groups were assessed by Fisher’s exact test. Statistical analysis was performed using SPSS software (version 22.0; IBM, Armonk, New York), and p < 0.05 was used as the threshold for significance.

## Results

### Demographics

The main clinical and radiological characteristics of all 380 patients are listed in **Table 1**. According to their clinical preoperative diagnose, the enrolled patients included 210 with epilepsy and 170 with no epilepsy. The dataset was randomly split into a training cohort (n = 266, epilepsy/no epilepsy = 147/119) and a testing cohort (n = 114, epilepsy/no epilepsy = 63/51).

**Table 1:** Patients’ clinical and radiological characteristics by univariate analyses for glioma-associated epilepsy grouping.

Characteristics	Training cohort (n=266)			Testing cohort (n=114)		
	Non-GAE group	GAE group	*p*-value	Non-GAE group	GAE group	*p*-value
**Sample size**	119	147	–	51	63	–
**Male/female**	57/62	90/57	0.030 *^b^*	25/26	44/19	0.024 *^b^*
**Age** Mean ± SD (range)	50 ± 12 (11–74)	44 ± 13 (12–75)	<0.001 *^a^*	50 ± 12 (17–73)	41 ± 12 (7–69)	0.002 *^a^*
**Glioma location 1** (Left/right/both)	48/57/14	77/63/7	0.039 *^b^*	19/11/21	30/4/29	0.055 *^b^*
**Glioma location 2**			0.026 *^b^*			0.379 *^b^*
Frontal lobe	44	69		24	29	
Occipital lobe	9	5		2	2	
Parietal lobe	13	24		6	7	
Temporal lobe	29	26		7	10	
Insular lobe	6	6		4	4	
Thalamus	7	0		1	0	
Lateral ventricle	4	1		3	0	
Multilobe	7	16		4	11	
**Glioma grade** (WHO II/III/IV)	26/22/71	78/29/39	<0.001 *^b^*	10/10/31	32/14/16	<0.001 *^b^*
**Glioma genotype**			<0.001 *^b^*			<0.001 *^b^*
IDH_w_/IDH_m_ 1p19q intact/IDH_m_ 1p19q codeletion	80/21/18	52/42/51		37/7/7	23/20/19	
**Involved cortex**			0.014 *^b^*			0.129 *^b^*
Edema/tumor/both/none	1/97/0/21	3/133/2/9		2/40/0/9	3/56/1/3	
**Cystic**			<0.001 *^b^*			0.001 *^b^*
Yes/no	93/26	68/79		41/10	32/31	
**Necrosis**			0.073 *^b^*			0.674 *^b^*
Yes/no	97/22	106/41		39/12	46/17	
**Edema degree**			<0.001 *^b^*			0.097 *^b^*
No/mild/moderate/severe	5/46/41/27	8/92/34/13		3/24/10/14	4/38/15/6	
**Hemorrhage**			<0.001 *^b^*			0.279 *^b^*
Yes/no	38/81	18/129		15/36	13/50	

IDH_w_, IDH wild type; IDH_m_, IDH mutation type.

p value < 0.05 was considered as a significant difference.

a Two-sided t-tests.

b Pearson chi-square test.

In all cohorts, there was a significant difference in sex between the GAE and non-GAE groups (male/female = 134/76, 82/88; p = 0.003), with male patients with glioma having a higher risk of epilepsy. Another basic clinical feature, patient age, was also significantly different between the GAE and non-GAE groups [(43 ± 13)/(50 ± 12), mean ± SD, p < 0.001], and according to the clinic-radiological model, age had a higher negative coefficient (-2.78) second to tumor location; thus, younger patients had a higher risk of epilepsy.

### Performance of the Clinic-Radiological Model

**Figure 2:**
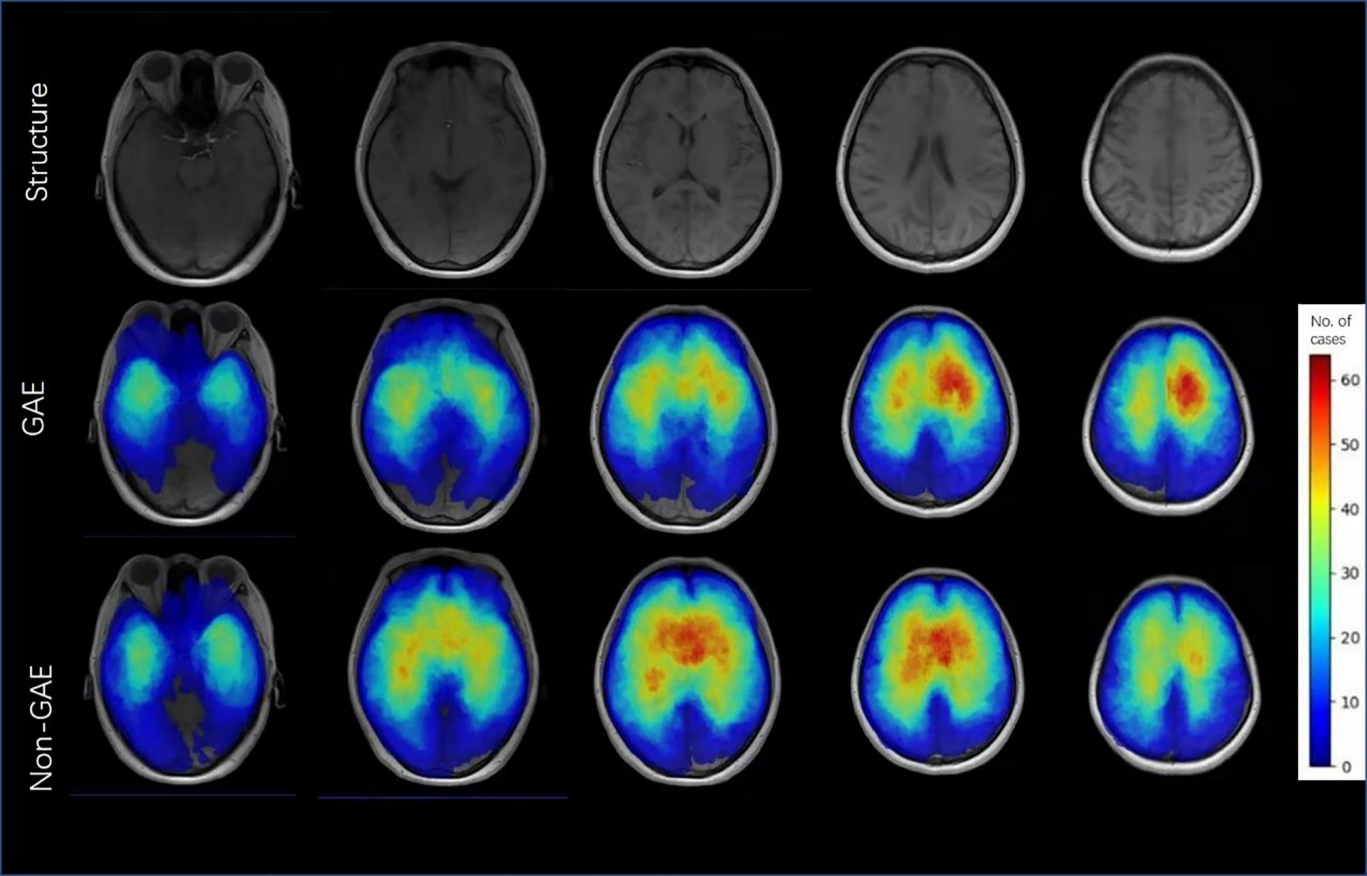
Color frequency map illustrates the location of and number of patients with glioma-associated epilepsy and non-GAE. Images are displayed in neurologic display convention.

There were significant differences between the GAE and non-GAE groups with respect to age, sex, glioma grade, and glioma type (**Table 1**). Younger, male, and LGG patients had a higher risk of experiencing GAE (**Table 1**). Regarding radiological characteristics, the GAE group was more likely to have no cystic components or hemorrhage. Furthermore, the tumor location of the non-GAE group tended to be close to the anterior commissure, and glioma involving the bilateral brain lobes was associated with a lower risk of GAE. Tumors in the GAE group tended to be in the left hemisphere, especially in the left frontal lobe (**Figure 2**).

**Figure 3:**
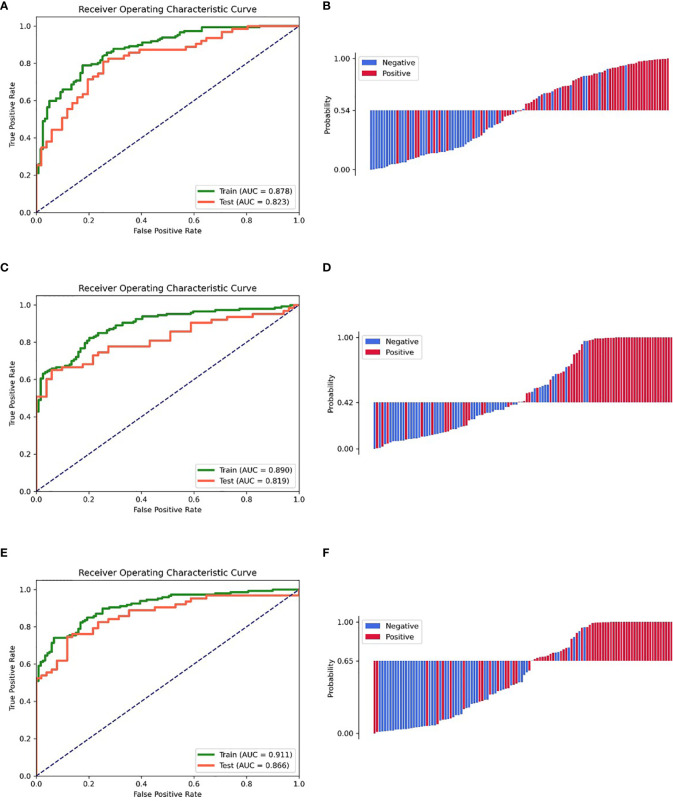
ROC curves of the training and testing cohorts, the waterfall plot of the distribution of prediction probability on the testing cohort. **(A, B)** Clinic-radiological model. **(C, D)** Radiomic signature. **(E, F)** Combined model.

The clinic-radiological model for GAE prediction is listed in **Table 2**, with AUC values of 0.878 and 0.823 for the training and testing cohorts, respectively. The sensitivity, specificity, and accuracy of the clinic-radiological model are summarized in **Table 3**. ROC curves and waterfall plots are shown in **Figures 3A, B**.

**Table 2:** Selected features and the coefficients of features in the clinic-radiological and radiomic signature for predicting glioma-associated epilepsy.

Clinic-radiological model		Radiomic signature	
Features	Coefficients of LR	Features	Coefficients of LR
Originated in the parietal lobe	5.676631	Wavelet LHL GLRLM run variance	6.383190937
Originated in the frontal lobe	5.367907	Wavelet HLH GLCM ICM2	4.798755709
Originated in the occipital lobe	4.79728	Wavelet HHL GLSZM gray-level variance	-4.1547
Originated in the insular lobe	4.568124	Wavelet LHL GLSZM small area low gray level emphasis	3.80425
Originated in the temporal lobe	4.502959	Original first-order kurtosis	3.548193
Originated in the lateral ventricle	2.970133	Wavelet HHL GLDM low gray-level emphasis	-2.73834
Age	-2.78469	Wavelet HHH first-order kurtosis	2.392724
Edema involved cortex	1.932897	Wavelet LHL GLSZM gray-level non-uniformity	-2.34516
Edema degree	-1.73233	Original GLRLM long-run high gray-level emphasis	2.183297
Cyst	-1.60373	LoG sigma 3.0 mm 3D GLDM low gray-level emphasis	1.643264
Hemorrhage	-1.33371	Wavelet HHL GLCM correlation	1.414969
Pathological grade	-1.12885	Wavelet HHL large dependence low gray-level emphasis	-1.40935
Bilateral brain lobes involved	-0.76147	Wavelet LHL GLCM correlation	1.199208
Left hemisphere involved	0.692024	Wavelet HLL GLCM correlation	0.966634
Tumor involved cortex	0.608529	LoG sigma 3.0mm 3D GLDM dependence non-uniformity normalized	-0.77698
Both edema and tumor involved cortex	-0.59825	Wavelet LHL GLSZM low gray-level zone emphasis	0.613669
Necrosis	0.479448	Original GLSZM low gray-level zone emphasis	-0.50718
Gender	-0.40985		
Originated in the thalamus	-0.3556		
Right hemisphere involved	0.069447		

GLCM, gray-level co-occurrence matrix; GLDM, gray-level dependence matrix; GLRLM, gray-level run length matrix; GLSZM, gray-level size zone matrix; HLL, HHL, LHL, HLH considering L and H to be a low-pass (i.e., a scaling) and a high-pass (i.e., a wavelet) function; LoG, Laplacian-of-Gaussian; ICM2, informational measure of correlation 2.

**Table 3:** The performance of all models in predict glioma-associated epilepsy in training and testing cohort.

Model	Cohort	AUC (95% CI)	Accuracy	Sensitivity	Specificity	PPV	NPV
Clinic-radiological model	Training	0.878 (0.836–0.917)	0.805	0.789	0.824	0.847	0.780
	Testing	0.823 (0.739–0.895)	0.745	0.714	0.784	0. 804	0.690
Radiomic signature	Training	0.891 (0.852–0.924)	0.812	0.823	0.798	0.835	0.785
	Testing	0.820 (0.733–0.893)	0.754	0.746	0.765	0.797	0.709
Combined model	Training	0.911 (0.878–0.942)	0.827	0.742	0.933	0.932	0.745
	Testing	0.866 (0.790–0.929)	0.798	0.746	0.863	0.870	0.733

AUC, area under the curve; PPV, positive predictive value; NPV, negative predictive value.

### Performance of the Radiomics Signature

A prediction model utilizing logistic regression was constructed by integrating the 17 key radiomic features (**Table 2**) obtained with the training cohort. The AUC, sensitivity, specificity, and accuracy of the radiomic signature are presented in **Table 3**. ROC curves and waterfall plots are shown in **Figures 3C, D**.

**Figure 4:**
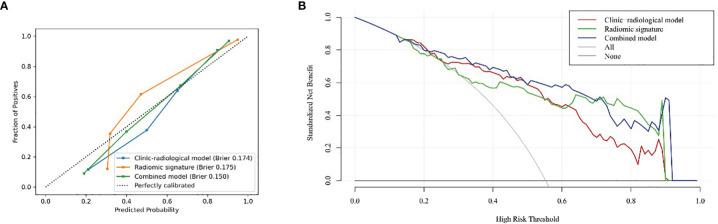
Calibration curve of testing cohort **(A)** and DCA curves **(B)**.

The radiomic signature demonstrated favorable calibration with the training and testing cohorts (**Figure 4A**). The p-values of the Hosmer–Lemeshow test for the predictive classification ability of the radiomic signature were 0.264 and 0.017, respectively. DCA showed that using the radiomic signature to predict epilepsy type added more benefit than either the treat-all-patients scheme or the treat-none scheme (**Figure 4B**).

### Performance of the Radiomics Nomogram

**Figure 5:**
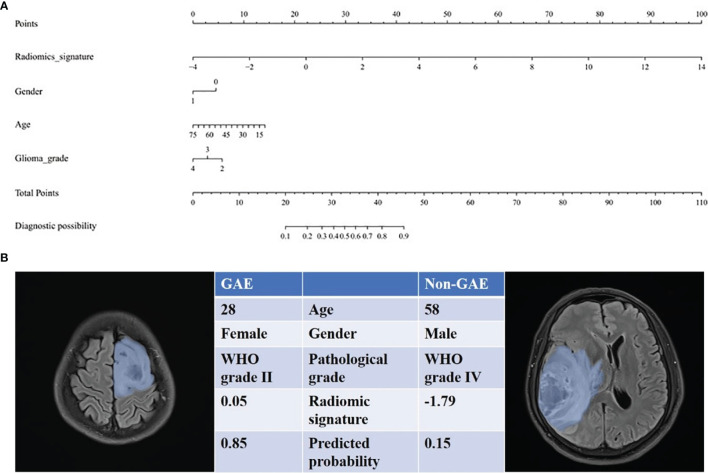
The radiomics nomogram for prediction of epilepsy type. **(A)** The radiomic-based nomogram was built using radiomics signature, age, gender (0 = male, 1= female), and tumor grade data. **(B)** Two cases for which the diagnostic probability of GAE was calculated.

The radiomics signature, age, sex, and tumor grade were used in the development of the combined model, which reached the highest AUC (training cohort, 0.911 [95% CI, 0.878–0.942]; testing cohort, 0.866 [95% CI, 0.790–0.929], **Table 3** and **Figures 3E, F**) among all the models. The DeLong tests indicated that the difference between the clinic-radiological model and the combined model in the testing cohorts (p = 0.207) was not statistically significant, as shown in **Table 4**. The radiomics nomogram for visualizing the combined model is shown in **Figure 5**. The calibration curves of the radiomics nomogram demonstrated satisfactory agreement between the predictive and observational possibility of the occurrence of GAE for both the training and testing cohorts (p = 0.820 and 0.023, respectively, Hosmer–Lemeshow test).

**Table 4:** Comparison of the performance of models in the testing cohort.

Comparison	Cohort	McNemar test *^a^* (p value)	DeLong’s test *^b^* (p value)
Combine model vs. radiomic signature	Train	0.862	0.051
	Test	0.132	0.010
	All	0.547	0.001
Combine model vs. clinic-radiological model	Train	0.465	0.312
	Test	0.200	0.207
	All	0.782	0.135
Radiomic signature vs. clinic-radiological model	Train	0.622	0.360
	Test	0.855	0.937
	All	0.808	0.381

p value <0.05 indicated a statistically significant difference.

a Test for comparison the difference of accuracy.

b Test for comparison the difference of AUC.

## Discussion

This study developed a quantitative and individualized combined radiomics model for predicting seizures in patients with WHO grade II–IV glioma using T2 FLAIR radiomic features relevant to GAE. To the best of our knowledge, this is the first study to construct a clinic-radiological model for GAE prediction in patients with WHO grade II–IV grade glioma (19–22). The results demonstrate that the T2 FLAIR-based combined radiomics model successfully stratified patients according to the occurrence of GAE. The radiomics nomogram, as an easy-to-use and powerful clinical tool, could be used to help clinicians assess the occurrence of GAE in patients with glioma.

Radiomics is said to reveal hidden patterns potentially associated with tumor phenotypes that are difficult to observe with the naked eye (27). However, the clinic-radiological model that used imaging signs detected by the naked eye demonstrated a performance in predicting GAE comparable to that of the radiomics model (p = 0.937). This result suggests that comprehensive and precise imaging signs identified by the naked eye from conventional MRI sequences are highly relevant in the diagnosis of GAE and should be utilized in the future, preferably in an automated manner. Although the model based on these imaging signs achieved similar performance as the radiomics model, identifying these signs required high reader expertise and workloads. Radiomics based on automatic segmentation, especially radiomic nomogram that combined radiomic features and simple clinical information, offers an easy, precise, convenient, and objective tool for clinicians to assess the occurrence of GAE in patients with glioma.

Although glioma located in any part of the brain may be associated with GAE, those located in the parietal and frontal lobes were linked to a higher risk of seizures, which is consistent with previous studies (1, 28). Tumors involving the bilateral brain lobes always coexist with wide white matter breaking, which limits the origination and propagation of epileptic discharges. Thus, tumors involving the bilateral brain lobes, especially those close to the anterior commissure, have a lower risk of GAE, which is consistent with the findings by of Yang (29), Akeret (30), and Lee (31). A higher risk of GAE was associated with glioma in the left hemisphere, especially when the left frontal lobe was involved, which is inconsistent with Akeret (30), Lee (31), and Polin (32) but consistent with studies from Yang (29) and Huang (33). This inconsistency may be attributed to the relatively small size of our sample or to ethnic differences.

In regard to the imaging signs, patients with glioma with peritumoral edema involving the cortex, a lower edema degree, no cystic components, and no hemorrhage had a higher risk of experiencing GAE. The region with peritumoral edema shows changes in the metabolism of the microenvironment; in particular, the pH is significantly elevated when peritumoral edema involves the cortex, which could cause seizures to arise electrographically from this region (34). A lower edema degree indicates a lower glioma grade; in particular, WHO grade II LGG has a greater association with GAE than high-grade glioma (HGG) (35, 36). Thus, there is no contradiction between peritumoral edema involving the cortex and a lower edema degree. Cystic components and hemorrhage are typical signs of large, rapidly growing tumors and have been shown to be associated with lower rates of seizures than smaller and slower-growing tumors (1).

Three of the clinical characteristics were included in the radiomics nomogram: sex, age, and glioma grade. In particular, patient age has been shown to be related to GAE (20), and in our study, age was an important factor in the clinic-radiological model. Age, serving as an indicator of the complex changes that occur in humans over time, is an important factor in many diseases. LGG patients are mostly young adults and have a higher risk of GAE than HGG (3). Change of metabolism, neuronal plasticity, and homeostasis between neurotransmitters are all highly correlated with the occurrence of epilepsy. All these factors are influenced by the age of the patient (37–39) and the characteristics of gliomas (5, 40, 41). Thus, age may be combined with other factors that are recognized or unrecognized temporarily by researchers. Sex is a complex and comprehensive influencing factor, and this is the first study to describe sex differences in the incidence of GAE. Patients with epilepsy experience complex changes in hormone secretion (42) given the complex interaction between hormones and epilepsy. Although the incidence of glioma is different in men and women (43), how sex influences GAE remains not clear (44).

There are some limitations to the present study. First, patients were recruited from a single center, and a multicenter research with a larger sample size would be promising for developing a better prediction model. Second, a single sequence was used, and multiple sequences may improve model accuracy. Third, an epidemiological study with a large sample is needed to test and verify the association between GAE and patient age and sex.

In summary, the radiomics nomogram developed in this study predicted the occurrence of epilepsy in patients with glioma in a non-invasive, simple, and practical manner. Comprehensive clinic-radiological imaging signs detected by the naked eye have reasonable and non-discriminatory performance in the prediction of GAE with respect to radiomics models.

## Data Availability Statement

The original contributions presented in the study are included in the article/**Supplementary Material**. Further inquiries can be directed to the corresponding authors.

## Ethics Statement

The studies involving human participants were reviewed and approved by Ethics Committee of Scientific Research and Clinical Experiments of the First Affiliated Hospital of Zhengzhou University. The patients/participants provided their written informed consent to participate in this study.

## Author Contributions

BJ and GA: manuscript preparation, literature research, data acquisition, statistical analysis, and manuscript editing. YH: manuscript preparation, literature research, data analysis, and statistical analysis. WY: literature research, data analysis, and statistical analysis. ZG, WC, and WH: literature research and data acquisition. ZX and MX: data analysis and study design. YG, ZY, and CJ: study conception and design, manuscript review, and guarantor of integrity of the entire study. All authors contributed to the article and approved the submitted version.

## Funding

This study was funded by a public service platform for artificial intelligence screening and auxiliary diagnosis for the medical and health industry, Ministry of Industry and Information Technology of the People’s Republic of China (No. CEIEC-2020-ZM02-0103/03); National Natural Science Foundation of China (61731009); National Key R&D Program of China (2016YFC0106900); and Medical Tackling Problems in Science and Technology Plain Program of Henan Province, China (201702070).

## Conflict of Interest

The authors declare that the research was conducted in the absence of any commercial or financial relationships that could be construed as a potential conflict of interest.

## Publisher’s Note

All claims expressed in this article are solely those of the authors and do not necessarily represent those of their affiliated organizations, or those of the publisher, the editors and the reviewers. Any product that may be evaluated in this article, or claim that may be made by its manufacturer, is not guaranteed or endorsed by the publisher.
