# Supplementary material for: Radiomics Nomogram Improves the Prediction of Epilepsy in Patients With Gliomas
Source: Front Oncol. 2022 Mar 30;12:856359. doi: 10.3389/fonc.2022.856359 (PMC9007085; doi:10.3389/fonc.2022.856359)
Supplement: Supplementary file 1 [file DataSheet_1.docx]

**Appendix 1. The definition of semantic radiological characteristics**

Necrosis: A region that exhibited high-intensity T2WI signals, but low intensity on T1WI signals, and with an irregular border.

Cyst: A well-defined and rounded region which had an extremely high signal intensity on T2-W images but a low signal on T1-W images, with a thin, regular and smooth wall.

Hemorrhage: A region that exhibited low intensity T2WI signals, but high or slightly low intensity T1WI signals, and with a signal loss on DWI (b=1000). Microhemorrhages, intratumoural apoplexy, and old bleeding residues are defined as with hemorrhage.

Eg. Figure a, male, 65-year-old, Astrocytoma WHO grade Ⅲ, IDH mutation, 1p19q non-codeleted. The blue circle indicates cyst. Figure b, female, 50-year-old, Oligodendroglioma WHO grade Ⅱ, IDH mutation, 1p19q codeleted. The red star denotes necrosis. Figure c, female, 57-year-old, Glioblastoma, IDH wild type, the orange triangle denotes hemorrhage.


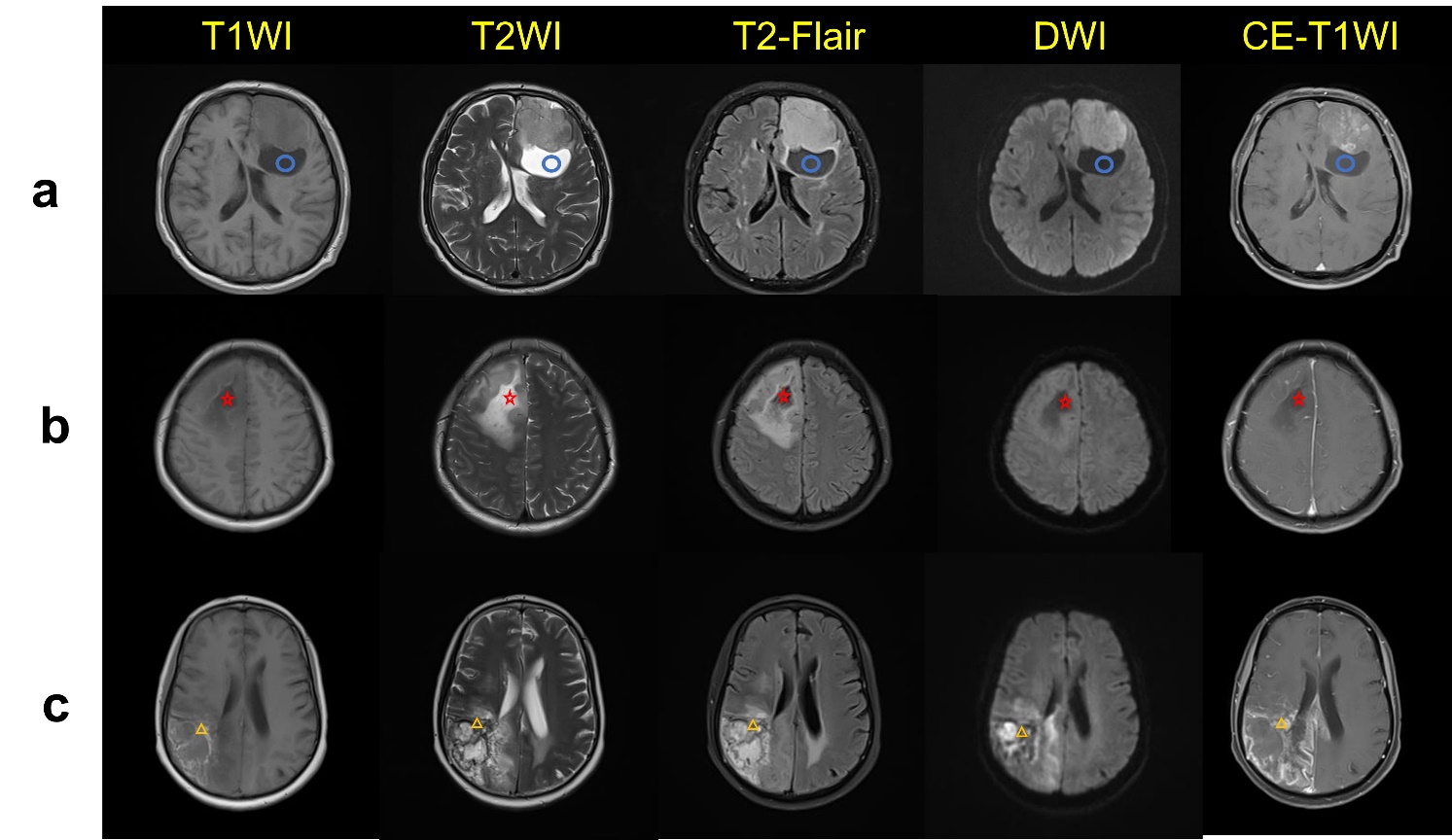


Edema degree: No edema, edema index=1; Mild edema, edema index=1~1.5; Moderate edema, edema index=1.5~3; Severe edema, edema index>3, (Edema index = (Volumes of edema + Volumes of tumor)/ Volumes of tumor.


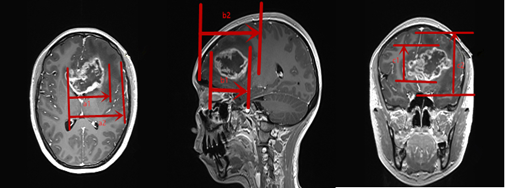
Eg. Male, 56year-old, Glioblastoma, IDH wild type. EI=（a2×b2×c2）/（a1×b1×c1）.
